# SUMO chain formation relies on the amino-terminal region of SUMO-conjugating enzyme and has dedicated substrates in plants

**DOI:** 10.1042/BCJ20170472

**Published:** 2018-01-02

**Authors:** Konstantin Tomanov, Lilian Nehlin, Ionida Ziba, Andreas Bachmair

**Affiliations:** Department of Biochemistry and Cell Biology, Max F. Perutz Laboratories, Center for Molecular Biology, University of Vienna, Dr. Bohr Gasse 9, Vienna A-1030, Austria

**Keywords:** *Arabidopsis thaliana*, AUX/IAA proteins, protein modification, SUMO, SUMO chains

## Abstract

The small ubiquitin-related modifier (SUMO) conjugation apparatus usually attaches single SUMO moieties to its substrates, but SUMO chains have also been identified. To better define the biochemical requirements and characteristics of SUMO chain formation, mutations in surface-exposed Lys residues of Arabidopsis SUMO-conjugating enzyme (SCE) were tested for *in vitro* activity. Lys-to-Arg changes in the amino-terminal region of SCE allowed SUMO acceptance from SUMO-activating enzyme and supported substrate mono-sumoylation, but these mutations had significant effects on SUMO chain assembly. We found no indication that SUMO modification of SCE promotes chain formation. A substrate was identified that is modified by SUMO chain addition, showing that SCE can distinguish substrates for either mono-sumoylation or SUMO chain attachment. It is also shown that SCE with active site Cys mutated to Ser can accept SUMO to form an oxyester, but cannot transfer this SUMO moiety onto substrates, explaining a previously known dominant negative effect of this mutation.

## Introduction

Sumoylation, the covalent addition of small ubiquitin-related modifier (SUMO) to substrate proteins, is an essential process in metazoa. In plants, sumoylation has been linked to development and stress responses [1–4]. Chromatin proteins, RNA processing factors and metabolic enzymes have been identified as substrates for SUMO attachment [5–9]. Consistent with a role in stress response, SUMO-conjugate levels rise transiently during stress [10]. To execute SUMO conjugation, heterodimeric SUMO-activating enzyme (SAE) links the SUMO carboxyl terminus to a Cys residue in the active site, from where activated SUMO is transferred to active site Cys of SUMO-conjugating enzyme (SCE). Substrate modification occurs at ε-amino groups of Lys residues, which are linked to the SUMO carboxyl terminus in an isopeptide bond. In contrast with the more complex ubiquitin-conjugation system, the sumoylation machinery consists of a surprisingly small number of components. One type of SAE and a single SCE are the core components in most organisms [1]. In particular, SCE can transfer SUMO to substrates without the participation of additional components. Affinity to the sumoylation consensus motif ψKxE (with ψ symbolizing an amino acid with hydrophobic side chain, x any amino acid, and K and E as single letter codes for Lys and Glu, respectively) directs SCE to substrates and leads to their sumoylation. The activity of SCE in SUMO transfer can be enhanced by factors of the SUMO ligase class that improve the alignment of SCE and substrate. The best-known SUMO ligases contain an SP-RING [Siz/PIAS (protein inhibitor of activated STAT)-RING (really interesting new gene)] domain as a docking site for SCE binding. Whereas most known substrates receive a single SUMO moiety, SUMO chains have also been detected *in vivo*, and their formation can be observed *in vitro*, using purified components SAE, SCE and SUMO. Similar to substrate decoration with single SUMO moieties, SUMO chain formation can also be enhanced by SUMO ligases [11,12]. To gain mechanistic insights into how SUMO chains are synthesized, we studied this reaction *in vitro*. We were particularly interested in finding mechanistic differences between mono-sumoylation of a substrate and SUMO chain formation. For this, we tested mutations in SCE for their effect on SUMO acceptance from SAE, on SUMO transfer to a substrate and on SUMO chain formation.

Mutations affecting the active site Cys of SCE abolish SCE function, for apparent reasons. However, there may be differences depending on the replacing residue. While a Cys-to-Ala change (C94A in Arabidopsis SCE1) results in an inactive protein, a Cys-to-Ser (C94S) mutant displays a dominant negative *in vivo* effect [13]. A Cys-to-Lys (C94K) change results in a protein that can be charged with SUMO by SAE. This latter mutant, charged with SUMO, has recently been used as a proxy for the unstable SCE–SUMO complex with thioester linkage to the active site, although geometric distances are not exactly the same [14,15]. Whereas the C94K version has been used mainly in structural biology, the C94S mutant was used *in vivo*, and its inhibitory effect was found both in animal cell culture and in plants [13,16]. Interestingly, the mode of action of the latter mutant has not been elucidated previously. In this work, we show that SCE C94S forms an oxyester bond with SUMO, explaining its dominant negative effect on sumoylation.

We have previoulsy described a mutation in the first alpha helix of SCE, a Lys 15-to-Arg (K15R) change, that decreases SUMO chain formation without a similarly large effect on mono-sumoylation of a model substrate [11]. To understand the basis of this effect, we tested and found additional mutants with similar discriminatory properties. These changes decrease SUMO chain formation, but still allow efficient transfer of SUMO moieties from SAE to SCE, and from SCE to substrates. We show that the mutations have a similar effect when chain formation is enhanced by SUMO ligase PIAL2. We also identified a substrate that is modified by SUMO chains *in vitro*, implying that whether a sumoylation substrate is modified by a single residue, or by a SUMO chain, is critically influenced by substrate properties. Finally, *in vivo* expression of the SCE K15R mutant revealed an inherent flexibility of cellular SUMO conjugation. Plants exclusively relying on SCE K15R do not show the reduced growth rate characteristic of decreased capacity for mono-sumoylation, but a slightly higher resistance to salt stress suggests lower rates of chain formation, consistent with the *in vitro* properties of the mutant enzyme.

## Materials and methods

### Vector construction

Vectors for expression of Arabidopsis SCE1 (briefly called SCE in the following) variants in *Escherichia coli* were generated by site-directed mutagenesis with primers listed in Supplementary Table S1. SCE K15R was created as described in Tomanov et al. [11]. SCE K15R was further mutagenized to generate SCE K15,19R, SCE1 K15,19,28R and SCE K15,19R K28Q. SCE WT was mutagenized to generate SCE K28Q, SCE K28R and SCE K146,147,150,153R. SCE C94S was described in ref. [13]. Additionally, SCE WT was mutagenized to create C94A and C94K. Mutagenesis was performed using the Kapa HiFi polymerase (Kapa Biosystems) according to the manufacturer's protocol, but for 18 cycles only. PCR products were digested with DpnI to remove the methylated template DNA and transformed into chemically competent *E. coli* XL1-blue cells as described previously. Vectors for expression of PIAL2, SAE and SUMO tag3 were described in refs. [5,11]. An N-terminally tagged SUMO (Flag-His) was created by excising the SUMO tag 3 with NcoI and Acc65I, then ligating the vector with complementary oligonucleotides possessing matching sticky ends. Thus, the Strep-HA-His tag (tag3) was replaced by a Flag-His tag. To obtain a vector for *in planta* expression of SCE1, the genomic region of SCE1 (AT3G57870), comprising 1000 bp upstream of the first ATG codon and ∼300 bp downstream from the stop codon of the gene, was amplified with the Kapa Plant 3G polymerase (Kapa Biosystems) according to the manufacturer's protocol. The primers contained restriction sites for XhoI (5′) and SpeI (3′). After adding adenine overhangs using a Taq polymerase, the PCR product was ligated into a pCR2.1 TA cloning vector (Life Technologies) and transformed into *E. coli* XL1-blue. Plasmid DNA was digested with XhoI and SpeI and ligated into pGreen. The plasmid was transformed into *Agrobacterium tumefaciens* GV2260 pSoup. Arabidopsis plants heterozygous for the T-DNA insertion allele *sce1-5* (SALK_138741; ref. [17]) were transformed with the *Agrobacterium* strain and progeny was selected by spraying 2-week-old seedlings (on soil) with Basta (40 mg/l Basta and 0.01% Silwet). The pGreen vector bearing the wild-type genomic construct was mutagenized using Kapa HiFi to introduce the K15R mutation, and the variant vector was used for plant transformation as the WT construct.

### Protein purification

Proteins for *in vitro* enzyme reactions were expressed and purified as described [11,18]. For isothermal titration calorimetry (ITC), SCE was further purified using a MonoS (GE Healthcare) ion exchange column on an ÄKTA 900 FPLC machine (GE Healthcare). Buffer A consisted of 10 mM NaPO_4_, 10 mM NaCl, 1 mM dithiothreitol (DTT) or 2-mercaptoethanol (βME) (depending on the experiment), 20% glycerol, pH 6.5. Buffer B consisted of 10 mM NaPO_4_, 1 M NaCl, 1 mM DTT or βME (depending on the experiment), 20% glycerol, pH 6.5. SUMO1-Flag-His was purified by immobilized metal ion affinity chromatography using an Ni^2+^ resin (GE Healthcare). In addition, protein for ITC was further purified using a MonoQ (GE Healthcare) ion exchange column on an ÄKTA 900 FPLC machine. Buffer A consisted of 5 mM Tris, 1 mM DTT or βME (depending on the experiment), 20% glycerol, pH 7.4. Buffer B consisted of 5 mM Tris, 1 M NaCl, 1 mM DTT or βME (depending on the experiment), 20% glycerol, pH 7.4.

### *In vitro* sumoylation reactions and their quantification

For *in vitro* sumoylation, 20 µl reactions were used as described (for substrate sumoylation, see ref. [11]; for thioester formation, see ref. [13]). After SDS–PAGE and transfer to a PVDF membrane (Millipore), the membranes were probed with Strep-Tactin II conjugated to alkaline phosphatase (IBA, dilution 1 : 10 000), mouse anti-FLAG M2 (Sigma, dilution 1 : 10 000) or rabbit anti-SCE1 (ref. [13]; dilution 1 : 2000–1 : 5000). AP-conjugated Strep-Tactin was visualized using a mixture of 15 µl of 5% 5-bromo-4-chloro-3-indolyl phosphate (BCIP) (Gerbu) and 30 µl of 5% nitroblue tetrazolium (NBT) (Sigma) in 10 ml of alkaline phosphatase buffer (100 mM Tris, 100 mM NaCl and 5 mM MgCl_2_, pH 9.5). The membrane was incubated in the dark, and the reaction was stopped by rinsing the membrane with water. After drying, the membrane was scanned.

Primary antibodies were incubated with anti-mouse or anti-rabbit HRP-conjugated secondary antibody (GE Healthcare, dilution 1 : 10 000). For visualization, the membrane was incubated with 2 ml WesternBright Sirius (Advansta) for 5 min in the dark. Detection was performed using a ChemiDoc Touch system (Bio-Rad). Band intensities were quantified using Image Lab 5.1 (Bio-Rad) or ImageJ.

### Isothermal titration calorimetry

For isothermal titration calorimetry (ITC), proteins were dialyzed against SUMO buffer (20 mM Tris and 5 mM MgCl_2_, pH 7.4) at 4°C overnight. The dialysis buffer was thereafter filtered and used for the ITC run. SCE moieties were centrifuged using a Vivaspin column with 5 kDa cutoff (Sartorius) to increase their concentration to 60 µM. SUMO1-Flag-His was used at a concentration of 600 µM. ITC was performed using a MicroCal ITC 200 (Malvern). The program was set to 20 injections, the first one with 0.4 µl, and the subsequent ones with 2 µl of ligand. Injection duration was 2 s/µl, with 150 s spacing and a filter period of 5 s. The cell was kept at 25°C, reference power was set to 3, initial delay of 60 s and stirring speed of 750 rpm. Three hundred microliters of SCE1 were loaded into the cell, 40 µl of SUMO1 were loaded into the syringe.

### Electron microscopy

Reaction products of an *in vitro* SUMO chain synthesis were diluted to a final concentration of 50 µg/ml in spraying buffer, containing 100 mM ammonium acetate and 30% (v/v) glycerol, pH adjusted to 7.4. After dilution, the samples were sprayed onto freshly cleaved mica chips (Christine Gröpl, Austria) and immediately transferred into a BAL-TEC MED020 high vacuum evaporator (BAL-TEC, Liechtenstein) equipped with electron guns. The rotating samples were coated with 0.6 nm Platinum (BALTIC, Germany) at an angle of 4–5°, followed by 8 nm carbon (Balzers, Liechtenstein) at 90°. The obtained replicas were floated off from the mica chips, picked up on 400 mesh Cu/Pd grids (Agar Scientific, U.K.) and inspected in an FEI Morgagni 268D TEM (FEI, The Netherlands) operated at 80 kV. Images were acquired using an 11 megapixel Morada CCD camera (Olympus-SIS, Germany).

### Plant growth and its quantification

Seeds were sown on plates containing agar as gelling agent (0.8%), kept in a coldroom (8°C) for 3 days and subsequently incubated at 22°C under long day conditions (16 h light, 8 h darkness). Seedling fresh weight without roots was determined 21 days after sowing. Plants on soil were grown under standard conditions (21°C, 16 h light, 8 h darkness). After germination at higher density, and before the emergence of true leaves, seedlings of comparable size were transferred to single pots for later photography, to determine rosette leaf area.

### Statistical data evaluation

Data for Figure 8 were analyzed using the IBM SSPS statistics package two-sided *t*-test, and data for Figure 9 were analyzed by ANOVA test of the SPSS statistics package. Data of Table 1 were analyzed by a two-sided *t*-test, assuming that more than 10 data points contributed to the ensuing *K*_d_ value (cf. Supplementary Figure S1). With this assumption, the values for WT and K28R mutant differ at the 99% level (*P* < 0.01), and those between WT and K15R differ at the 99.9% level (*P* < 0.001).

**Table 1:** Affinity of SUMO for WT and mutant SCE as determined by ITC

SCE variant	*K*_d_ value (µM)
WT	710 ± 230
K15R	33 ± 17
K28R	270 ± 330

## Results

**Figure 1.**
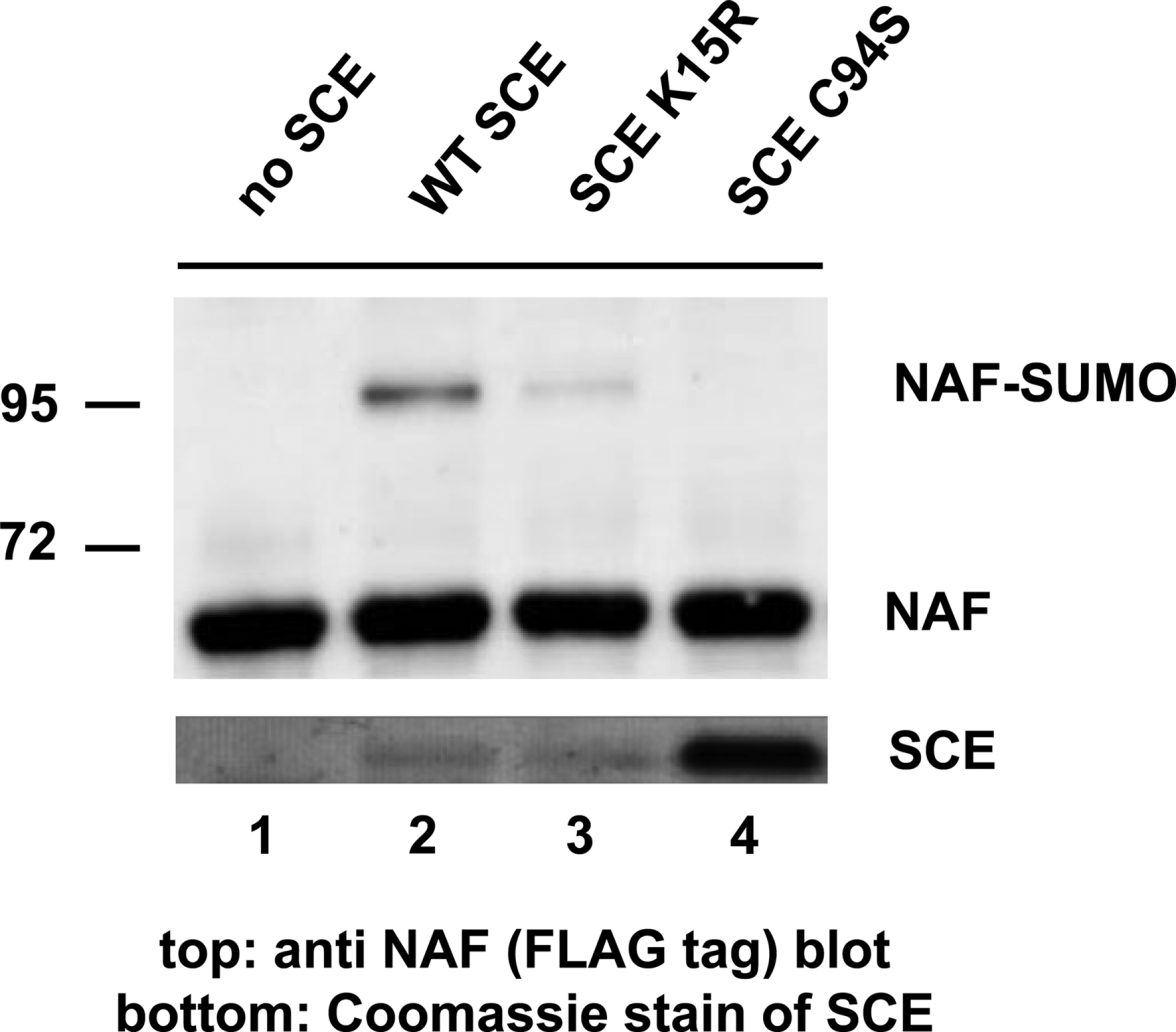
SUMO conjugation to model substrate NAF by WT SCE and SCE K15R. WT SCE and SCE K15R link one SUMO moiety to model substrate NAF. Active site mutant SCE C94S serves as negative control (10-fold excess of enzyme used).

**Figure 2.**
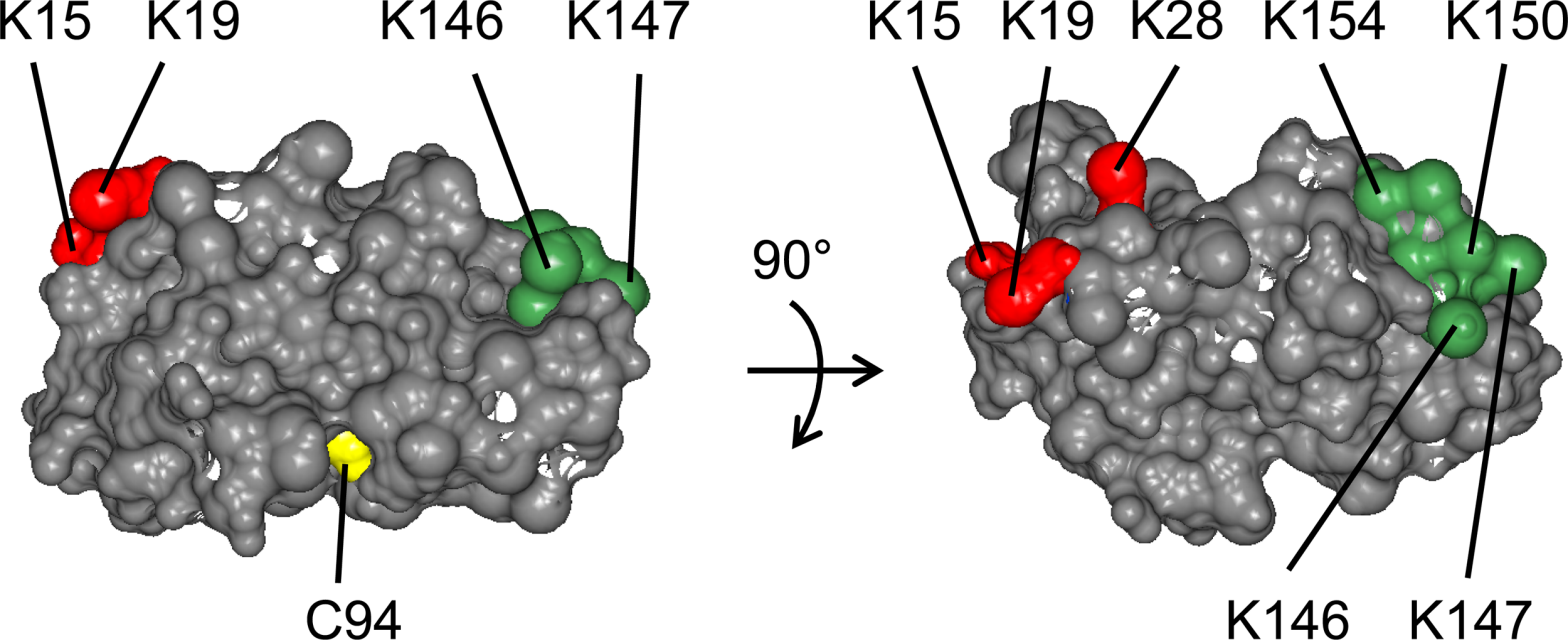
Side chains of Arabidopsis SCE1 mutated in this work. Mutations in the active site Cys residue (C94) abolish the activity of SCE, but some still allow loading with SUMO (see the text). Mutations in the first alpha helix (Lys residues 15, 19 or 28) have more impact on SUMO chain formation than on the mono-sumoylation activity of SCE. In contrast, mutation of Lys residues of the carboxyl-terminal region (Lys residues 146, 147, 150 and 154) to Arg has little impact on any activity of the enzyme.

SCE can *in vitro* link SUMO to a broad array of substrates, and it can also form SUMO chains. Arabidopsis SP-RING domain protein PIAL2 enhances SUMO chain formation by SCE [11]. Concomitant with increased appearance of SUMO chains, increased levels of SUMO-modified SCE were detected *in vitro*, implying that PIAL2 also catalyzes sumoylation of SCE. A mass spectrometry (MS) analysis indicated modification of SCE at Lys 15. As expected, the K15R mutation abolished SCE sumoylation by PIAL2. Figure 1 shows a slight decrease in the transfer of single SUMO moieties onto substrate nucleosome assembly factor (NAF) (At2g19480) by SCE K15R compared with WT SCE. Active site mutant SCE C94S, even in 10-fold excess, is incapable of SUMO transfer. Interestingly, in contrast with the rather mild effect on mono-sumoylation, the K15R mutation significantly decreased SUMO chain formation [11], suggesting a causal relationship between SCE sumoylation at K15 and chain synthesis. To confirm or disprove such a causal relationship, further mutagenesis of SCE was carried out by converting additional Lys residues of SCE into Arg. Figure 2 shows positions of residues modified in this work, which are surface-exposed Lys residues and the active site Cys.

**Figure 3.**
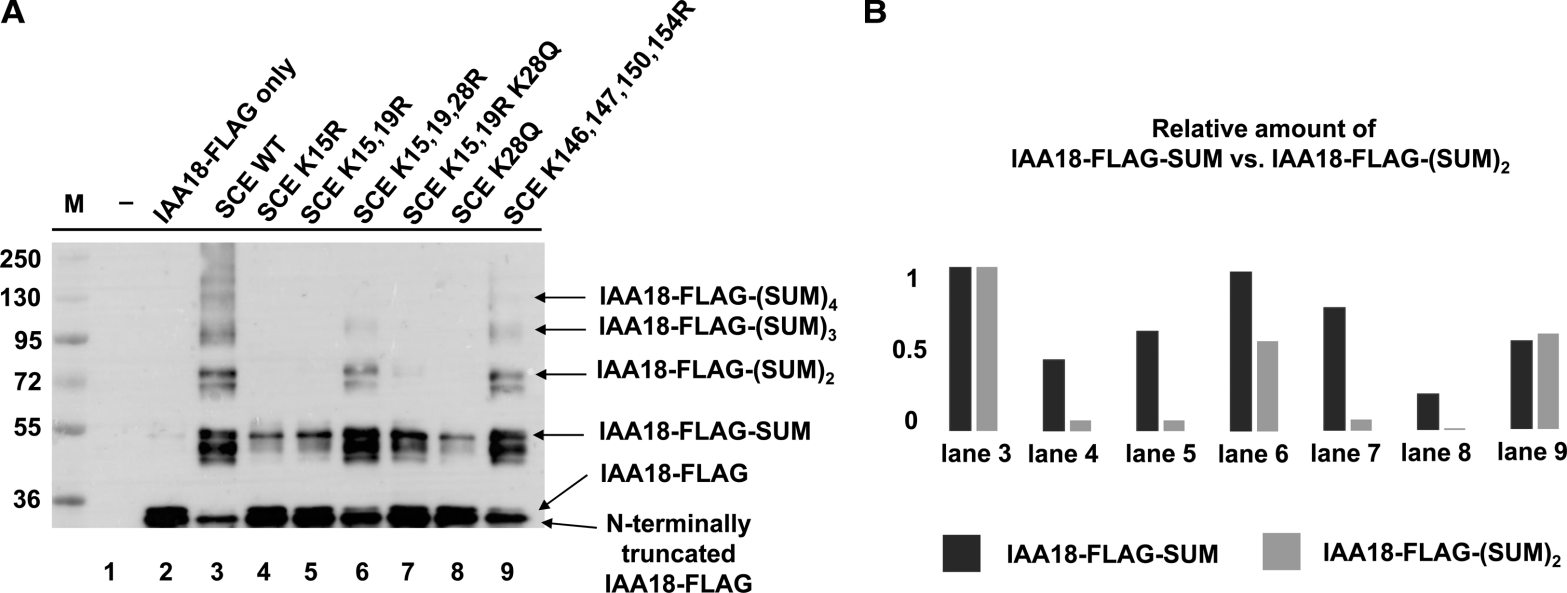
*In vitro* synthesis of SUMO chains on substrate protein IAA18 with different SCE variants. (**A**) Gel blot and (**B**) quantitation of the intensity of IAA18-SUMO versus IAA18-SUMO_2_. SCE variants K15R and K15,19R and K28Q are disproportionally affected in SUMO chain formation, when compared with the addition of the first SUMO residue onto IAA18. In contrast, the K28R mutation acts as an intragenic suppressor. K-to-R changes in the carboxyl-terminal region of SCE have little influence on SUMO chain formation, or on mono-sumoylation of IAA18.

*In vitro* sumoylation of substrate NAF (Figure 1) results in the addition of one SUMO moiety to the substrate. However, we present in this work a plant protein that is modified by conjugation of a SUMO chain. The substrate, IAA18 (At1g51950), belongs to a class of plant-specific transcriptional repressors. As shown in Figure 3, this substrate allows to test, in the same reaction mixture, the extent of mono-sumoylation versus SUMO chain formation by different SCE variants. Figure 3A shows a representative gel (of several replicates) and Figure 3B the quantitation of band intensities. The results are consistent with the results obtained by the assessment of free SUMO chain formation, and of mono-sumoylation of model substrate NAF, in separate reactions. Figure 3 supports the notion that the SCE K15R mutation reduces SUMO chain formation more than mono-sumoylation.

Based on the hypothesis that sumoylation of SCE K15 plays an important role in SUMO chain formation, it seemed possible that residual activity of the SCE K15R mutant might be due to SUMO modification of a nearby Lys residue. Thus, double-mutant SCE K15,19R was tested for its ability to synthesize SUMO chains. The ability of SCE K15,19R to form SUMO chains was also reduced to an extent roughly comparable with the K15R single mutant. Similar to SCE K15R, SCE K15,19R can still transfer single SUMO moieties to the substrate (Figure 3) with reasonable efficiency. A further mutant was generated, in which Lys 28 was also changed to Arg (SCE K15,19,28R). This triple mutant eliminated all Lys residues in the amino-terminal region of SCE. The activity of this mutant was surprising, though. The third mutation acted as a suppressor of the K15,19R changes. It is unlikely that a K28R change can compensate for the absence of a SUMO moiety on K15 or K19. We, therefore, concluded that the importance of K15 (or K19) for SUMO chain formation does not depend on the ability to be sumoylated, but that these residues (and K28) have a different function with relevance to SUMO chain formation.

Participation of sumoylated SCE in SUMO chain formation was previously postulated for the yeast SCE homolog, ubiquitin conjugating enzyme 9, the SCE homolog of animals and fungi (UBC9) (together with unmodified UBC9; ref. [19]). In this case, SUMO is conjugated to a Lys residue in the carboxyl-terminal region of UBC9. To exclude a similar case for plants, all Lys residues from the carboxyl-terminal region of SCE were replaced by Arg. A quadruple mutant Arabidopsis SCE K146,147,150,154R was able to form SUMO chains *in vitro*, at a rate comparable with WT SCE, and its ability to form chains and to mono-sumoylate a substrate were similar to WT (Figure 3A, lane 3 versus 9). Taken together, no indication was found for the involvement of SUMO-conjugated SCE in SUMO chain formation in plants.

**Figure 4.**
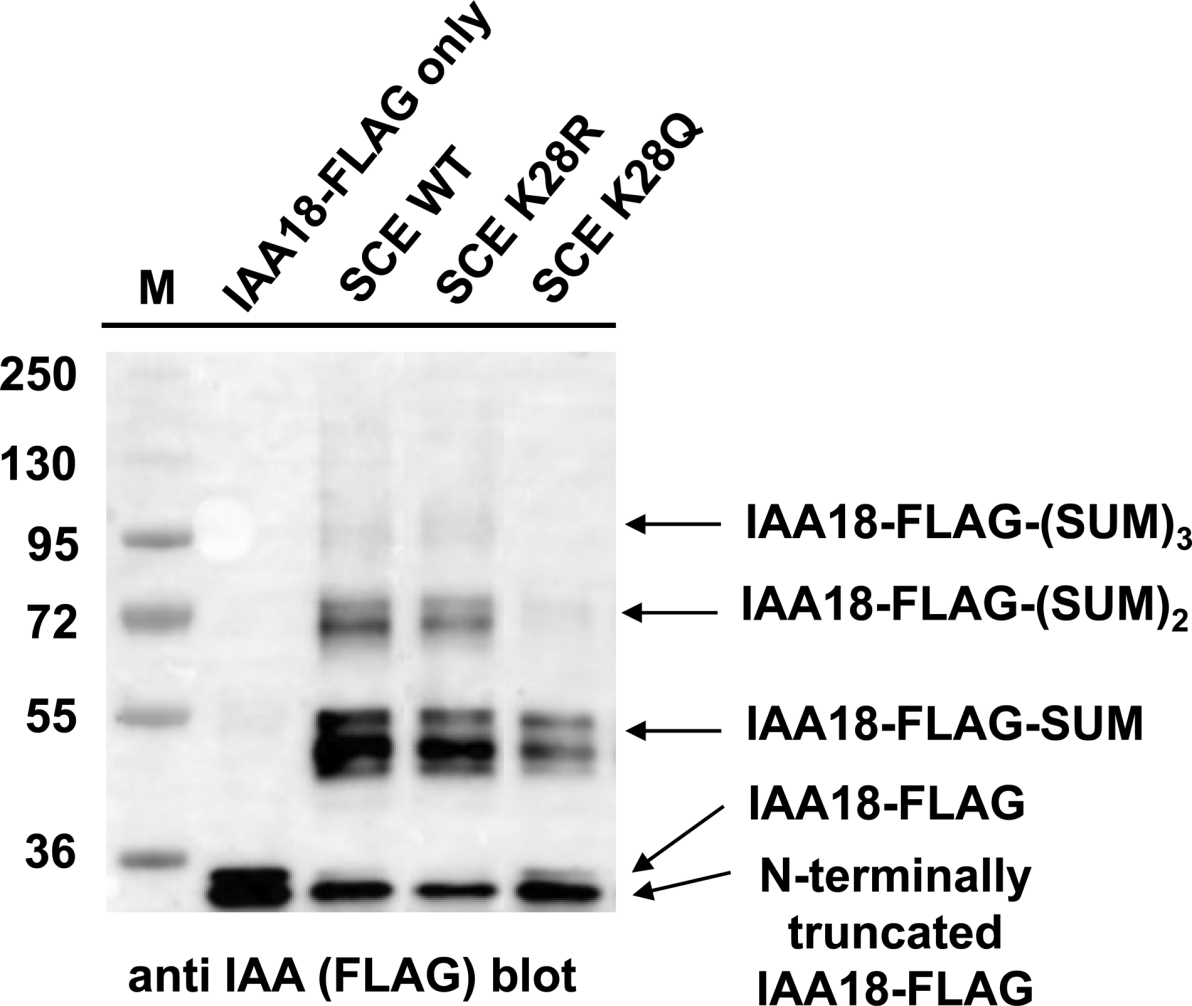
Single mutations SCE K28R and K28Q influence SUMO chain formation. The indicated SCE variants were used in reactions with substrate IAA18 to monitor both substrate sumoylation and SUMO chain formation.

If the surface encompassing Lys 15, 19 and 28 is an important interface with an impact on SUMO chain formation, then some changes of K28 might also interfere with this hypothetical interaction. The K-to-R change maintains charge, but results in a side chain that sticks out a little bit further due to the increased length of the Arg side chain compared with Lys. This size increase might strengthen the hypothesized interaction. In contrast, removal of the charge might have an opposite effect. Gln is polar but uncharged, and has a slightly decreased side chain length. A triple SCE K15,19R K28Q mutant and the single SCE K28Q mutant were tested. Both showed reduced ability to form SUMO chains, and similar to the K15R change, mono-sumoylation of the substrate is less severely affected (Figure 3A, lanes 7, 8). In contrast with the K15R and K15,19R mutations, the single K28R mutation in the WT SCE background has no inhibitory effect on SUMO chain formation (Figure 4). In many experiments, the mutation actually enhanced chain formation, but this enhancement apparently lies within the variability of the *in vitro* experimentation.

**Figure 5.**
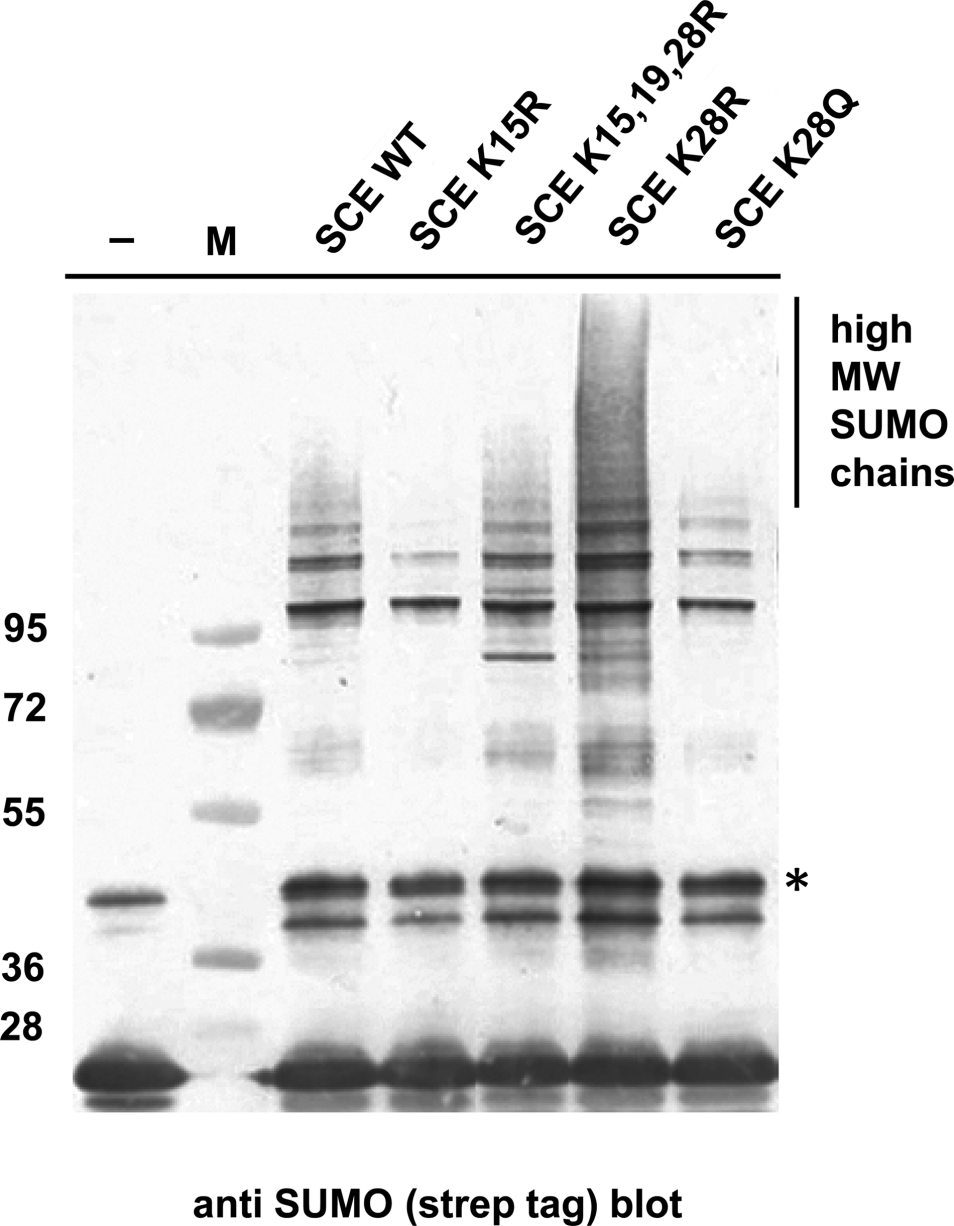
SUMO chain synthesis with SUMO chain-forming ligase PIAL2 depends on the same critical residues as SUMO chain formation by SCE alone. Reactions containing SUMO, SAE, SCE variants and a catalytically active fragment of SUMO chain-forming ligase PIAL2 were incubated to monitor SUMO chain formation. SUMO variants that fare poorly in assays without PIAL are also poor chain formers in the presence of PIAL2, and the K28R suppressor mutation also acts as a suppressor in the reaction with PIAL2. –, SUMO preparation used. M, molecular mass markers. Asterisk, contaminating band of the SUMO preparation.

The same SCE mutations that had been assayed for activity in the absence of SUMO chain-forming ligase PIAL2 were also tested in the presence of PIAL2. Figure 5 shows that all those mutants that fared poorly in the absence of PIAL2, also did so in its presence. Likewise, suppressor mutation K28R has a beneficial effect in the presence of PIAL2. Thus, the same interface of SCE is critical for SUMO chain formation with and without chain-forming ligase PIAL2. A tentative interpretation of this result is that SUMO chain formation in the presence or absence of PIAL2 proceeds via a similar mechanism.

**Figure 6.**
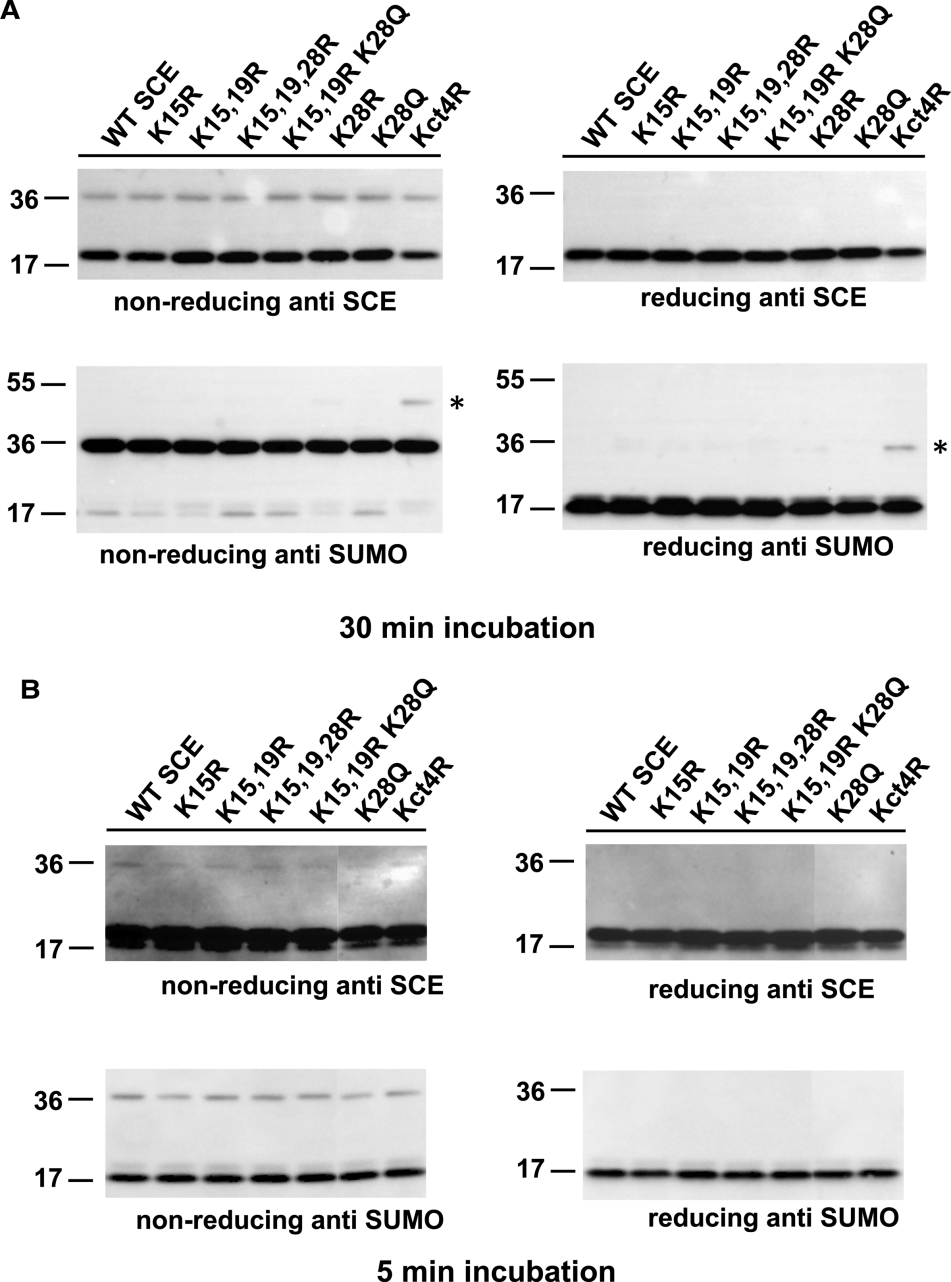
Thioester formation of SCE variants by the transfer of SUMO from SAE. (**A**) A 30 min incubation of SCE with SAE and SUMO allows detection of SCE–SUMO thioester. SUMO is almost completely consumed during this time. None of the SCE mutants differs in this end point assay. (**B**) Five-minute incubations under otherwise identical conditions do not uncover different kinetic parameters for SUMO transfer from SAE to SCE. Kct4R symbolizes SCE mutant with four Lys residues of the carboxyl-terminal end replaced by Arg (at positions 146, 147, 150 and 154). Asterisks, unidentified bands. The SCE–SUMO thioester band runs close to the 36 kDa marker band. For 5 min reaction of SCE K28R, see Supplementary Figure S2.

To make a SUMO chain, SCE has, to execute, several association–dissociation cycles with SAE, whereas substrate mono-sumoylation requires only one such cycle. To rule out that differential activities of chain building versus mono-sumoylation reflect a decreased ability to accept SUMO from SAE, we carried out thioester formation experiments for all SCE variants. Figure 6 shows no significant differences among the SCE variants. We, therefore, concluded that, under our *in vitro* conditions, formation of the SCE–SUMO thioester is not rate-limiting, so that the observed differences in SUMO chain formation may result from changes after SAE–SCE interaction. Structural data defining the SAE–SCE interaction surface [20] are consistent with this result. While the first alpha helix of SCE forms an interface with the ubiquitin-fold domain (UFD) of SAE, K15 points away from the interface. K19 is at the end of alpha helix one and may not be involved in SAE binding either. K28 is in the turn between two short beta strands (β1 and β2). This turn flexibly aligns with the UFD. It is, therefore, plausible that a conservative K-to-R change does not interfere with SAE binding.

Previous results had indicated that SUMO chain formation in animals and in fungi has a requirement for non-covalent binding of SUMO to SCE [21–23]. This binding occurs at the ‘back side’ of SCE, an interface opposite to the active site. Assuming that the same is true in plants, two SCE mutants tested for activity were assayed for non-covalent SUMO binding. ITC measurements were applied (Table 1 and Supplementary Figure S1). Under the conditions used, *K*_d_ for non-covalent binding of SUMO to Arabidopsis WT SCE is 710 ± 230 µM. SCE variants were tested that carried the K15R or the K28R mutation. Surprisingly, while these two mutants have opposing effects on chain formation, their effects on backside binding deviate from WT in the same direction. Both show stronger non-covalent SUMO binding. The *K*_d_ of the K15R mutant is more than 20-fold lower (33 ± 17 µM), while the measured value for SCE K28R is lower by only a factor of two (270 ± 330 µM; this difference is still statistically significant at the 99% level; see Materials and Methods). It is worth noting, however, that backside binding of SUMO to animal UBC9 seems much stronger [23,24], so that the *K*_d_ values for plant SCE K15R and SCE K28R are still significantly higher than those published for animal UBC9.

**Figure 7.**
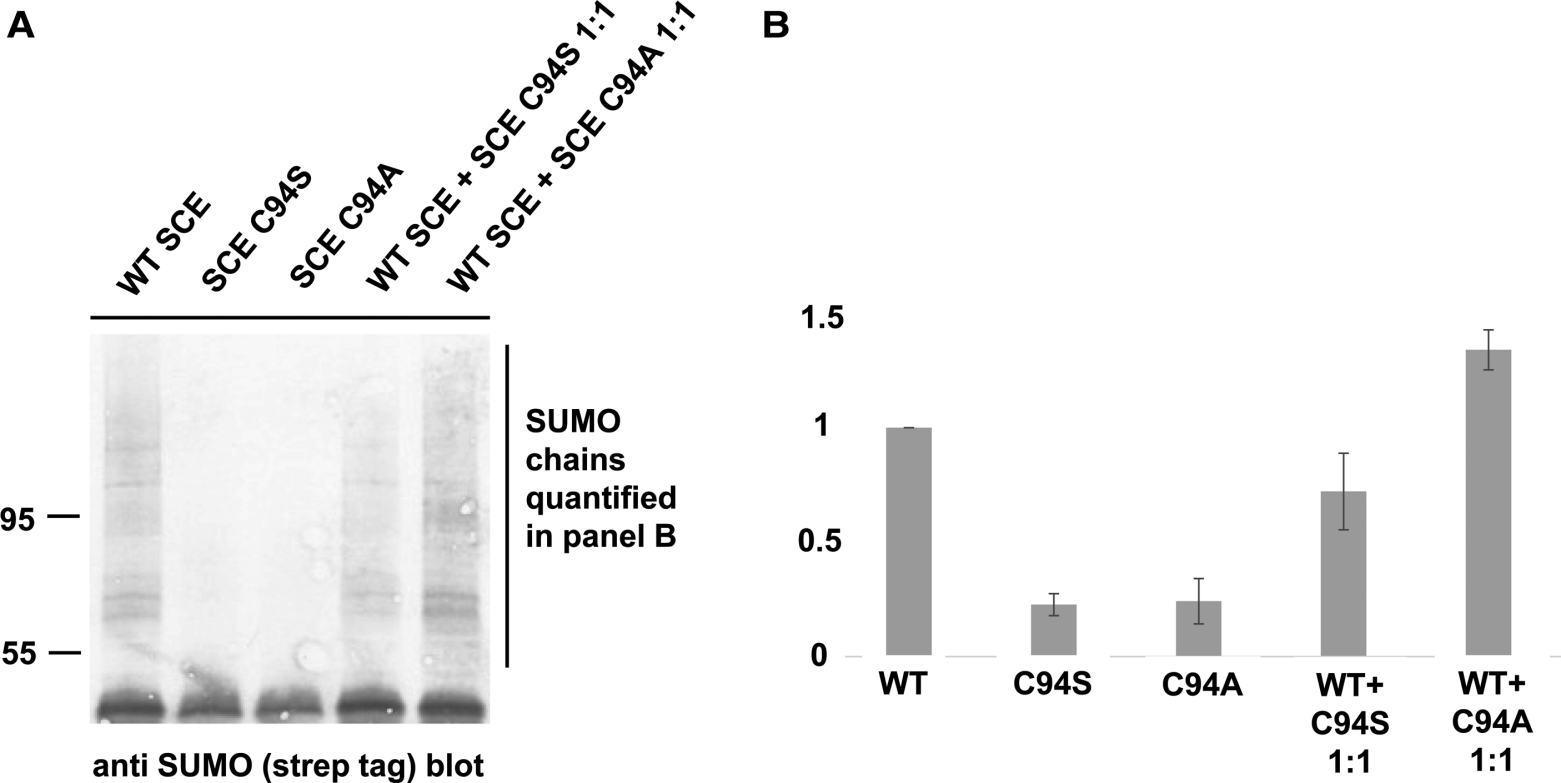
SUMO chain formation is slightly enhanced by the addition of equimolar amounts of SCE C94A. In an *in vitro* reaction consisting of tagged SUMO, SAE and SCE, the addition of an equal amount of SCE C94A, but not of SCE C94S, slightly enhances SUMO chain formation. (**A**) Representative gel. (**B**) Quantitation of SUMO chain abundance (average of three gels, amounts relative to WT).

Independent of possible covalent modification of SCE by SUMO, or of non-covalent SCE–SUMO binding, plant SCE engaged in SUMO chain synthesis may be part of a larger complex that contains an additional SCE moiety. We, therefore, tested how equimolar addition of SCE C94S or SCE C94A influenced SUMO chain formation (Figure 7). The addition of SCE C94A resulted in a slight increase in SUMO chains by ∼1.34-fold (±0.17; *P* = 0.06 in a two-sided *t*-test; Figure 7 shows one representative gel), whereas SCE C94S decreased SUMO chain formation. In contrast, the addition of a 10-fold excess of SCE with mutation in the active site decreases SUMO transfer for both SCE C94S and SCE C94A (Supplementary Figure S3). Based on the effect of SCE C94A, it seems possible that SUMO chain formation engages a second SCE protein without SUMO transfer function. The effect of SCE C94S is apparently dominant negative.

**Figure 8.**
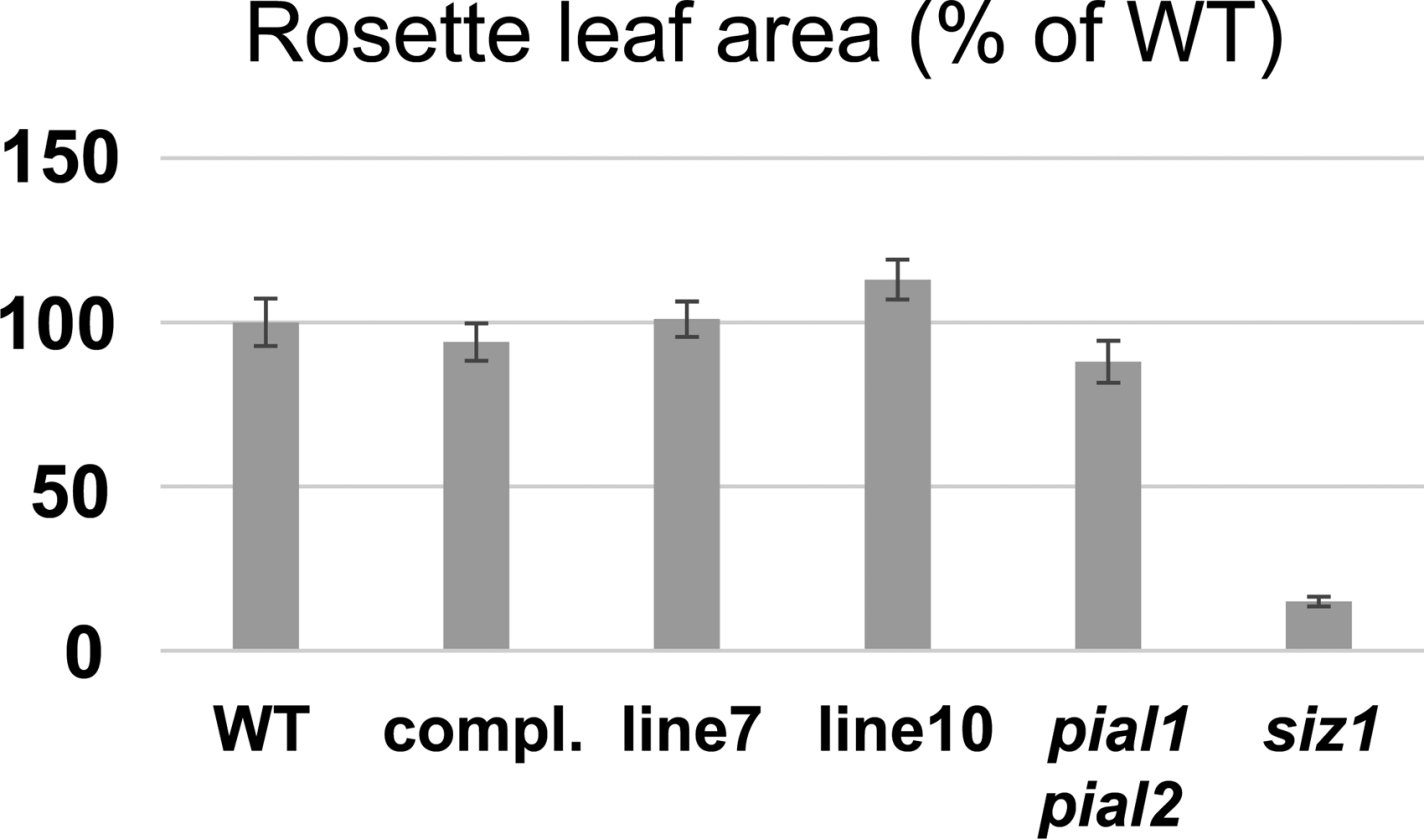
Growth of rosette leaves of plants with different lesions in SUMO conjugation. Plants were grown in single pots and photographed when WT rosettes were ∼6 cm in diameter. Leaf area was determined using ImageJ (value ± SEM). ‘compl.’ means that an *sce1-5* homozygous mutant was complemented by the introduction of a WT SCE transgene, and lines 7 and 10 contain an SCE K15R transgene in the *sce1-5* background. Only *siz1* mutant growth is significantly reduced (*P* < 0.001, two-sided *t*-test, compared with all other genotypes).

**Figure 9.**
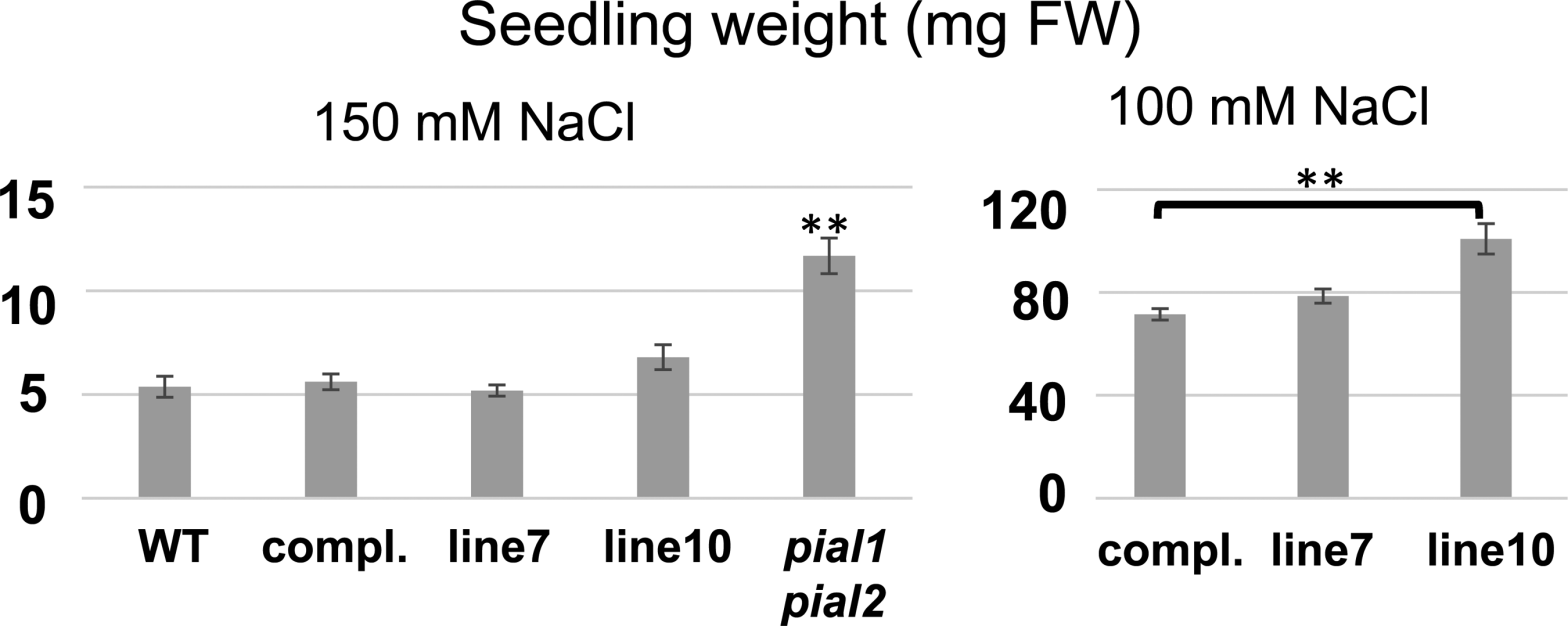
Growth of seedlings on plates containing NaCl. Plants were grown on plates with the indicated NaCl concentration for 3 weeks before fresh weight was determined. Values are mean ± SEM. ‘compl.’ indicates a WT SCE gene complementing the *sce1-5* mutation, and lines 7 and 10 contain an SCE1 K15R transgene in the same background. In the left panel, only growth of *pial1 pial2* seedlings is significantly different from all other samples. In the right panel, growth of the complemented line (compl.) differs significantly from line 10, but not from line 7 (** indicates *P* < 0.001, two-sided *t*-test; FW indicates fresh weight).

We also tested SCE K15R *in vivo*. Plants hemizygous for the loss-of-function *sce1-5* T-DNA insertion allele (the mutation is lethal in the homozygous state; ref. [17]) were transformed with a construct containing a genomic fragment that consists of promoter, coding region and terminator of the Arabidopsis SCE1 gene, with the K15R mutation. The same construct with the WT SCE sequence served as a control. Progeny were selected for homozygosity of the *sce1-5* allele and for good expression of the SCE K15R copy. Two lines with SCE K15R were chosen for further analysis. In one assay, growth of rosette leaves was tested, because three different methods of decreasing SUMO-conjugation capacity *in vivo* were previously shown to result in reduced growth [13,25,26]. Figure 8 shows average rosette leaf area ± SEM. Mutants defective in the SIZ1 SUMO ligase were used as an example for reduced growth. As previously found, the *pial1 pial2* mutant (deficient in SUMO chain-forming ligases PIAL1 and 2) showed normal growth. Neither *sce1-5* plants supplemented with the WT SCE construct, nor those supplemented with SCE K15R, showed significant deviation from WT growth. We concluded that the K15R mutation does not grossly disturb sumoylation processes under non-stress conditions. Another assay was growth on medium containing NaCl. Previous work had indicated that SUMO chains are necessary for proper response to salt stress, and *pial1 pial2* mutants display higher resistance to NaCl. At a salt concentration of 100 mM NaCl, there was a small, but significant effect for one SCE K15R line (Figure 9). The other line also showed an effect, but it was not statistically significant. These results may suggest a decrease in SUMO chain formation for the SCE K15R mutant *in vivo*. However, this assumption has to be confirmed by direct measurement of SUMO chain levels.

**Figure 10.**
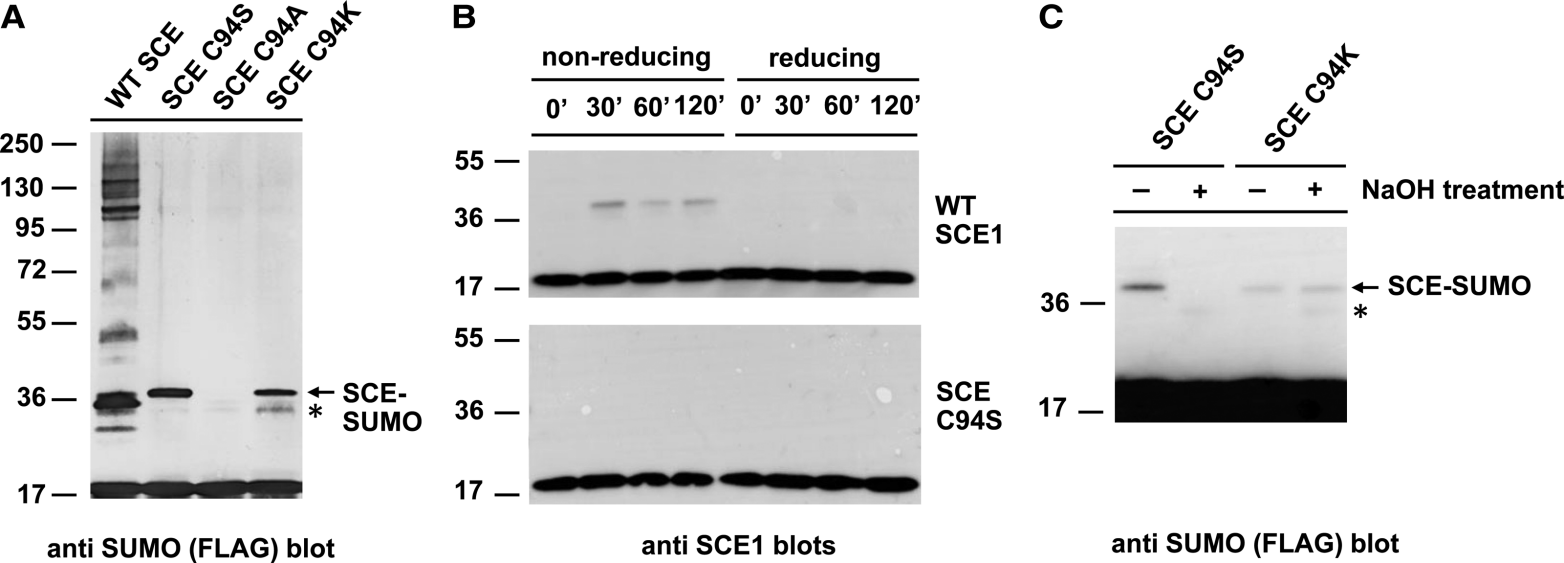
SCE C94S can form an active site oxyester with SUMO. (**A**) Upon extended reaction times under conditions that favor substrate sumoylation (or SUMO chain formation), SCE C94S and SCE C94K, but not SCE C94A, form an SCE–SUMO adduct that is stable under reducing conditions. (**B**) WT SCE (top), but not SCE C94S (bottom), can bind SUMO in a thioester linkage under conditions favorable for the reaction of WT SCE (left part, non-reducing). The reaction proceeds quickly. Reducing condition (sample boiling with DTT) destroys the linkage (right part, reducing). (**C**) The SCE–SUMO adduct with SCE C94S, but not that with SCE C94K, is sensitive to alkali treatment, indicating an oxyester linkage between the two proteins. The asterisk indicates a contaminating band of the SUMO preparation.

Under reaction conditions optimized for substrate sumoylation, anti-SUMO antibody revealed an additional band on analytical gels with SCE C94S, but not with SCE C94A. The size of the band was suggestive of a covalent SCE C94S–SUMO complex. The covalent addition of SUMO onto SCE with an active site Cys changed to Lys has been reported [14,15], so that an SCE C94K mutant was included in another set of reactions without a substrate. The SCE C94K mutant resulted in an identically sized band as SCE C94S (Figure 10A). This result was surprising, because SUMO loading of SCE C94S by SAE had not previously been detected. An earlier experiment [13], in which reaction conditions favored SUMO transfer from SAE to SCE, was repeated and the resulting thioester was resolved on a gel under non-reducing conditions. As previously published, SCE C94S was not loaded with SUMO under conditions that generated the WT SCE–SUMO thioester adduct (Figure 10B). This contrasts with the conditions optimized for substrate sumoylation and longer incubation times, under which SCE C94S was loaded with SUMO (Figure 10A). To rule out that SUMO is linked to a Lys residue adjacent to the active site in this reaction, a patch of three Lys residues next to the active site groove of SCE was mutated (Supplementary Figure S4). The triple Lys 72, 75, 77 to Arg change did not abolish the formation of an SCE C94S–SUMO conjugate (Supplementary Figure S5). In contrast with the SCE C94K–SUMO bond, the SCE C94S–SUMO bond is alkali-sensitive, as expected for an oxyester (Figure 10C). We, therefore, concluded that the dominant negative effect of SCE C94S is exerted by an overly stable SCE–SUMO adduct to active site Ser that is incapable of further SUMO transfer. Because this adduct forms much slower, and its formation *in vitro* is promoted by slightly different reaction conditions than the corresponding thioester of WT SCE, it has not been detected *in vitro* so far. These results explain why SCE C94S, *in vitro* and *in vivo*, influences sumoylation reactions in a way that differs from SCE C94A.

We finally addressed a question regarding the structure of SUMO chains. MS analyses from different laboratories [11,27] had indicated that SUMO–SUMO linkages are not exclusively formed onto a single Lys residue. However, two of the three Lys residues identified as SUMO attachment sites reside in the flexible amino-terminal extension of SUMO, which is conserved in canonical plant SUMO isoforms such as Arabidopsis SUMO1 and 2 [28]. Several possible SUMO attachment sites mean that SUMO chains could be branched. To resolve this issue, we subjected *in vitro* generated SUMO chains to electron microscopy. Chain synthesis included chain-forming ligase PIAL2. Spreads were processed by rotary shadowing. The expected structure of ∼4 nm dots (for the SUMO core fold) separated by 4 nm spacers (consisting of the flexible amino-terminal extension of one SUMO moiety linked to the carboxyl-terminal di-Gly motif of another SUMO, which also extends from the folded core) could be frequently identified. Supplementary Figure S6 shows that SUMO chains have an extended conformation on spreads. Because no images of branched chains were obtained, we concluded that SUMO chains are predominantly linear in structure.

## Discussion

In animals and in plants, SUMO ligases have been identified that specifically promote SUMO chain formation [11,12]. The existence of these enzymes, and the conservation of the motif used for SUMO attachment in the SUMO amino-terminal flexible extension (for plants, see ref. [28]), strongly suggests that SUMO chains possess physiological importance, and indeed, Arabidopsis plants with defect in SUMO chain-forming ligases show differences in stress response compared with the WT, as well as changes in protein and phosphoprotein abundance [11,29]. Nonetheless, SCE alone can *in vitro* both synthesize SUMO chains and mono-sumoylate substrate proteins.

Experiments of this work were initiated to better understand the mechanism of SUMO chain formation in plants, and how it differs from the addition of single SUMO moieties to substrates. SCE mutants with changes in surface-exposed Lys residues were analyzed for their impact on SUMO chain formation. All of these showed the basic functionality to mono-sumoylate a model substrate (Figures 1, 3 and 4). An assay for SCE–SUMO thioester formation confirmed that the SCE interaction with SAE is also not grossly perturbed by the mutations tested (Figure 6). However, most mutations decreased SUMO chain formation. We interpret this result as indication that SUMO chain formation relies, in part, on interactions that differ from those important for thioester formation or substrate sumoylation. Furthermore, the *in vitro* ability of mutant SCEs to form SUMO chains changed to the same extent with or without SUMO chain-enhancing ligases, suggesting that plant ligases that enhance SUMO chain formation build on a basic reaction executed by SCE alone, and do not operate via a distinct, entirely novel mechanism. Such a model is also consistent with structural data available for a SUMO chain-forming ligase (fragment) from humans in complex with UBC9 [14].

Starting point for the investigation was the previous finding that SCE with a Lys 15 to Arg (K15R) change displays significantly decreased ability to form SUMO chains (both with and without additional ligase). Because Arabidopsis SUMO chain-forming ligase PIAL2 promotes SUMO conjugation to SCE K15, we had previously assumed that enhancement of SUMO chain formation and SCE sumoylation are mechanistically linked, in that SCE K15-SUMO may be part of a SUMO chain-forming complex. However, we found that mutation SCE K28R (Lys 28 is located close to K15 on the SCE surface; Figure 2) acts as a suppressor of the K15R change. Because it is unlikely that the K28R change can compensate for the absence of a SUMO moiety in its vicinity (linked to K15), we concluded that K15 sumoylation and SUMO chain formation are independent from each other. The sumoylation of SCE on K15 by PIAL2, while not necessary for SUMO chain formation, may change substrate specificity of SCE as part of an integrated stress response executed by PIAL2. Incidentally, besides the two functions of SCE sumoylation and SUMO chain enhancement, PIAL2 has yet another function, as subunit of a transcriptional repressor complex [30].

Interestingly, sumoylation of animal UBC9 on a residue comparable with (Arabidopsis) SCE Lys 15 has been reported. This latter modification apparently promotes SUMO conjugation to substrates with a suitably located SUMO interaction motif, leading to altered affinities in the SUMO substrate space [31]. An extended role for SUMO-modified animal UBC9 in SUMO chain formation was not reported. In contrast, SUMO chain formation by yeast UBC9 was reported to require sumolyation close to its carboxyl-terminal end [19]. We also tested an SCE mutant devoid of Lys residues in the carboxyl-terminal region (Figure 2). The ensuing enzyme can form both free and substrate-anchored SUMO chains, indicating that SUMO chain formation by plant SCE does not require SUMO conjugation to a Lys residue close to its carboxyl terminus (Figure 3).

SCE with a K28R mutation was found to be at least as active in SUMO chain formation as the WT enzyme, and more active in the presence of ligase PIAL2 (Figures 4 and 5), whereas the K15R and the K28Q mutant showed severely reduced activity (Figures 3–5). All enzymes are still capable of decorating a model substrate with a single SUMO moiety. K15 and K28 may, therefore, participate in interactions that are more important for SUMO chain formation than for substrate mono-sumoylation. SCE has affinity for binding SUMO on a surface opposite to the active site Cys [21–23]. This interaction was previously shown to be essential for SUMO chain formation. Consistent with this, the structure of a domain from a SUMO chain-forming ligase in complex with SCE contains a SUMO moiety bound to the SCE back side [14]. While the exact role of this ‘backside binding’ for SUMO chain formation is still unclear, the interaction does not involve SUMO's carboxyl terminus, suggesting that not only free SUMO, but also SUMO bound via its carboxyl terminus to another SUMO residue or to a substrate, may be able to interact with SCE. We tested whether SCE K15R and SCE K28R show changes in their affinity to SUMO. The results of Table 1 indicate that both have increased affinity for non-covalent binding of SUMO. The magnitude of the effect differs, though, with SCE K15R deviating more from the WT than the K28R mutant. It should be noted that back side binding affinity between animal SUMO and UBC9 was reported to be ∼1000-fold higher, so that both *K*_d_ values measured for the SCE mutants are still above the value for the interaction in animals [23,24]. Nonetheless, we cannot rule out that a decrease in *K*_d_ could contribute to decreased SUMO chain formation by SCE K15R, and to increased SUMO chain formation by SCE K28R, in the SCE K15,19R background or in the presence of SUMO ligase PIAL2 (Figures 3 and 5). However, we favor the interpretation that the (opposite) effects of K15R and K28R mutations on SUMO chain formation result from their impact on yet another interaction necessary for SUMO chain formation.

We also investigated whether SUMO chain formation employs more than one SCE moiety, with mechanistically distinct roles. In particular, one SCE might have a largely structural function and thus not require its active site (it may, however, use its SUMO-binding surface to promote the reaction). In that case, one might expect that substrate sumoylation and SUMO chain formation show differential sensitivity to mixtures of mutant and WT SCE, or that trans-complementation of different SCE mutants is possible. Mutant SCE variants that contain changes in the active site were employed. When added in excess, these mutant proteins inhibit substrate sumoylation (Supplementary Figure S3). However, an equal amount of SCE with active site Cys-to-Ala mutation actually slightly enhances chain formation (Figure 3), suggesting the involvement of a second SCE protein in chain formation. However, resolution of this issue requires structural information on active complexes. Incidentally, in characterizing active site mutants of SCE, we could find an explanation for the dominant negative activity of the SCE C94S mutant (the active site in Arabidopsis SCE is in position 94). The C94S variant forms an oxyester with SUMO, which is more stable than the normal thioester (Figure 10). The loaded SCE may displace the WT enzyme from relevant complexes, resulting in a dominant negative effect *in vivo* [13,16] and *in vitro* (Supplementary Figure S3).

Plants relying exclusively on SCE K15R show normal growth (Figure 8). Because three distinct ways to decrease (mono-)sumoylation in Arabidopsis all reduce growth under normal greenhouse conditions [13,25,26], we concluded that the moderately decreased mono-sumoylation capacity of SCE K15R observed *in vitro* does not lead to a significant *in vivo* inhibition of this process. Interestingly, plants can significantly up-regulate SCE protein abundance in case of decreased sumoylation capacity [26,29]. This flexibility might contribute to the normal growth of SCE K15R plants. The *in vivo* model provides hints, but does not conclusively confirm a reduction in SUMO chain formation by exclusive expression of SCE K15R (Figure 9). From two independent transgenic lines, only one shows a robust phenotype of improved growth on NaCl (this phenotype was found in plants devoid of SUMO chain-forming ligases PIAL1 and PIAL2; [11]). The method of ectopic expression, as occurs via T-DNA transformation, may entail too many fluctuations in gene expression to allow more precise measurements. In any case, the *in vivo* results strengthen the tentative model that K15R and other conserved changes such as K28R do not significantly interfere with substrate mono-sumoylation. Taken together, we concluded that, for the SCE K15R mutation, *in vitro* results are by and large consistent with the *in vivo* effects.

It has previously remained unclear how substrates for SUMO chain addition are selected. Substrate selection for protein modifiers is usually the domain of ligases. *In vitro*, plant SUMO ligase PIAL2 and its homolog PIAL1 efficiently form free SUMO chains [11]. The addition of mono-sumoylation substrate NAF to a reaction containing SAE, SCE, SUMO and PIAL2 did not result in substrate poly-sumoylation [11]. The results of this work indicated that at least in a fraction of cases, decoration of a substrate with SUMO chains is determined by its interaction with SCE (Figures 1 and 3). Even without PIAL2 ligase, the protein AUX/IAA18 is modified by a SUMO chain *in vitro*. The protein had been detected as *in vivo* sumoylation substrate by an MS-based search for sumoylated proteins [5], but had not been analyzed extensively so far. The protein is part of the growth regulation in plants, whereby association of the plant hormone auxin with an F-box protein increases auxin-inducible gene family member (AUX/IAA) protein turnover via the respective SCF complex (Skip, Cullin, F-box containing complex) [32]. The ensuing decreased steady-state level of these co-repressor proteins changes the transcriptional pattern. It now seems possible that plants have a hormone-independent turnover route, via poly-sumoylation and SUMO-dependent ubiquitin ligases [33]. Interestingly, a route for by-passing normal hormone responses via sumoylation has recently been described for DELLA transcriptional repressor proteins, which change abundance in response to the plant hormone gibberellin [34]. Regarding AUX/IAA18, future research will show which properties of this protein trigger SUMO chain addition, and how widespread this degradation pathway is among the other members of the AUX/IAA protein family.

While it is unclear to which extent sumoylation reactions in a cell are carried out by SCE without SUMO ligases, this work shows that *in vitro*, a minimal system consisting of only SAE and SCE can carry out substrate mono-sumoylation and SUMO chain formation on a substrate. It would be surprising if this broad spectrum of activities of SCE would exist without being employed *in vivo*.

## Abbreviations

βME: 2-mercaptoethanol
AUX/IAA: auxin-inducible gene family member
DTT: dithiothreitol
ITC: isothermal titration calorimetry
MS: mass spectrometry
NAF: nucleosome assembly factor
SAE: SUMO-activating enzyme
SCE: SUMO-conjugating enzyme
SP-RING: Siz/PIAS (protein inhibitor of activated STAT)-RING (REALLY INTERESTING NEW GENE)
SUMO: small ubiquitin-related modifier
UBC9: ubiquitin conjugating enzyme 9, the SCE homolog of animals and fungi
UFD: ubiquitin-fold domain
