# Supplementary material for: SUMO chain formation relies on the amino-terminal region of SUMO-conjugating enzyme and has dedicated substrates in plants
Source: Biochem J. 2018 Jan 2;475(1):61–74. doi: 10.1042/BCJ20170472 (PMC5748838; doi:10.1042/BCJ20170472)
Supplement: Supplementary Figures and Table [file BCJ-475-61-s1.pdf]

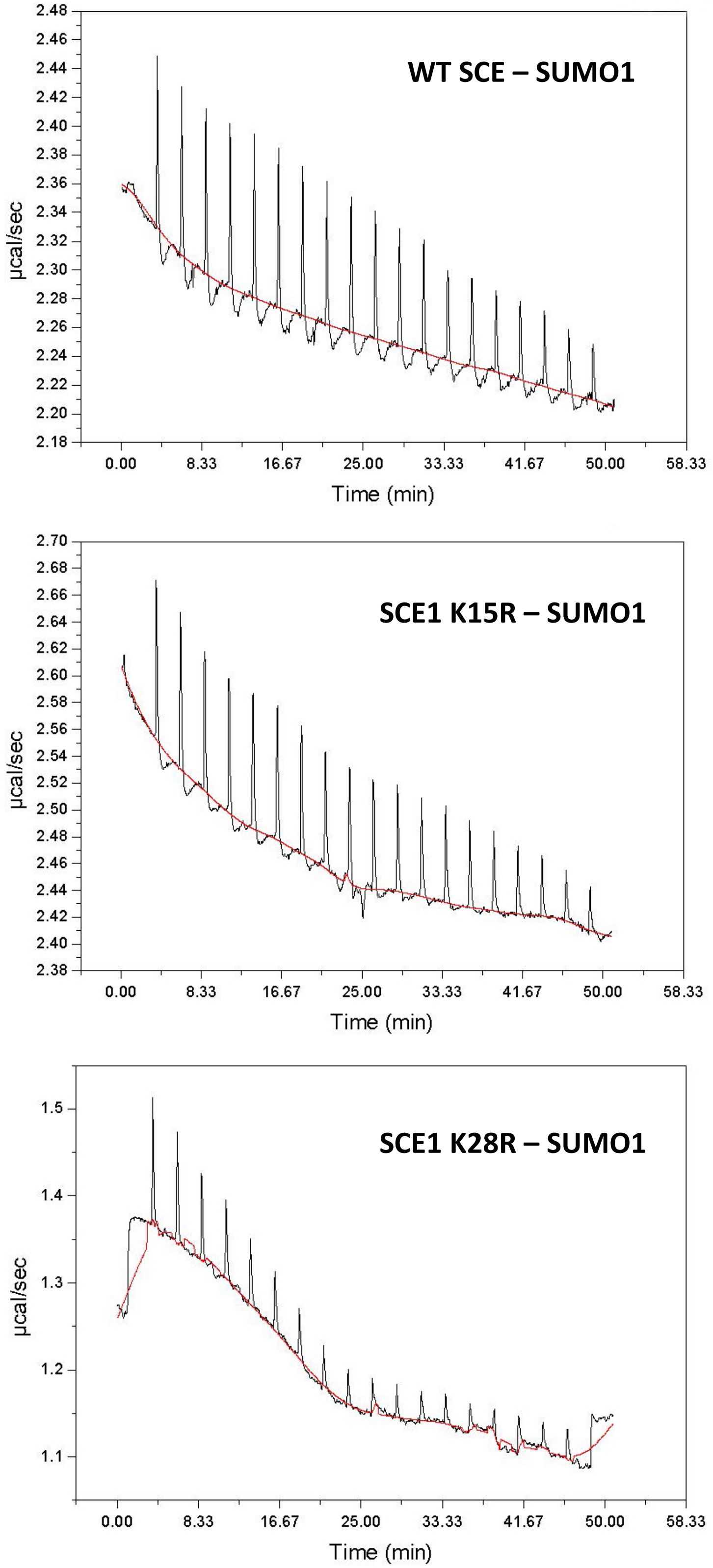

Supplementary Figure 1. Data readout of isothermal titration calorimetry. For details, see Materials and Methods section.

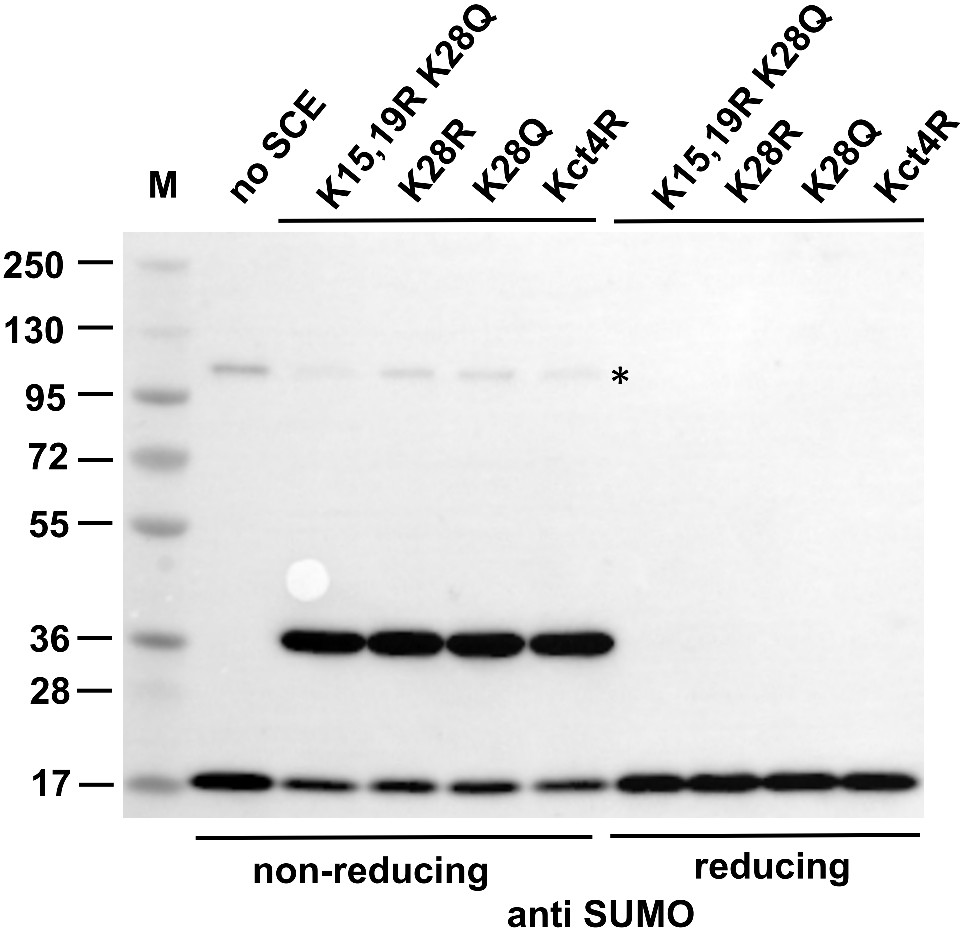

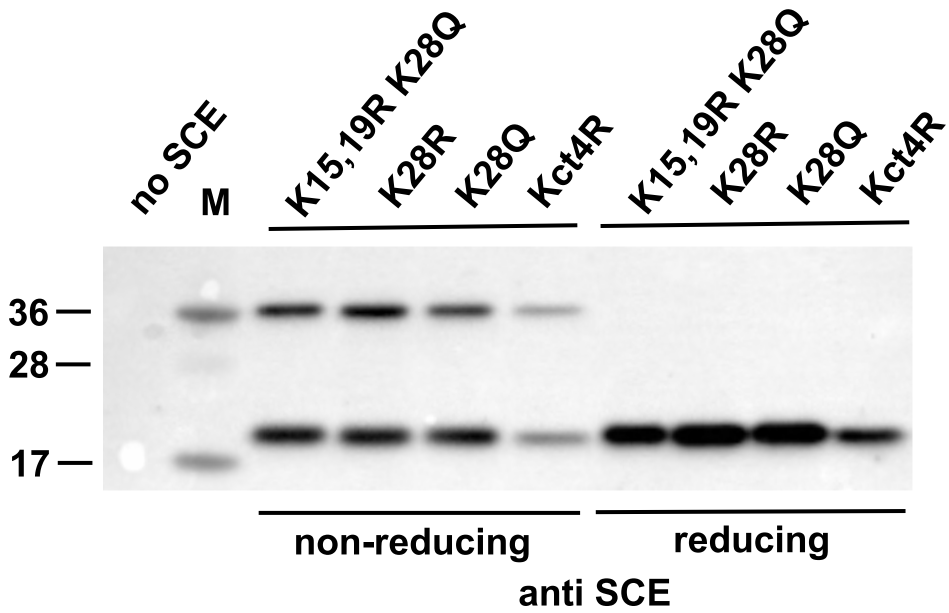

Supplementary Figure 2. Thioester formation of SCE variants by transfer of SUMO from SUMO activating enzyme SAE.

5 minute incubations under otherwise identical conditions do not uncover different kinetic parameters for SUMO transfer from SAE to SCE. Kct4R symbolizes SCE mutant with four Lys residues of the carboxyl-terminal end, at positions 146, 147, 150 and 154 replaced by Arg. See also Fig. 6 for more SCE variants. The top band in the top panel (asterisk, non-reduced reactions) is the SAE2-SUMO thioester. The SCE-SUMO thioester band runs at ca. 36 kDa.

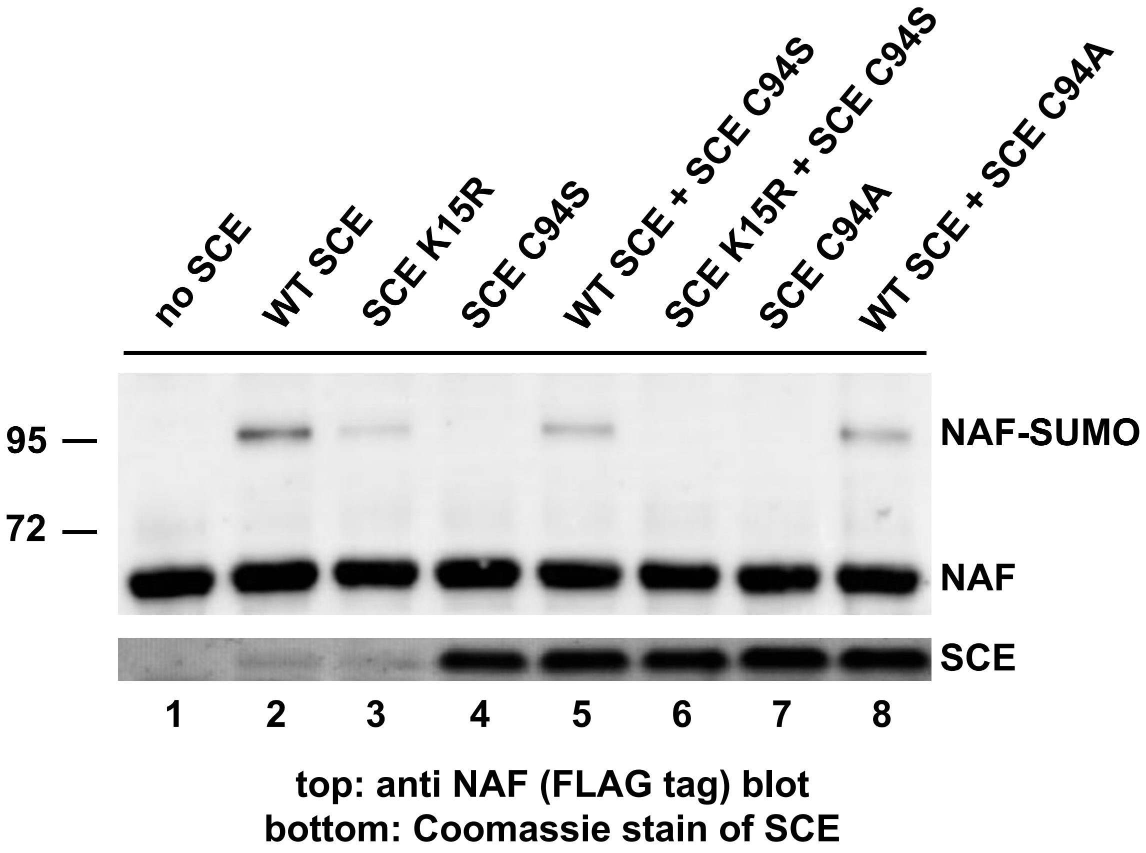

Supplementary Figure 3. SUMO conjugation to model substrate NAF by WT and mutant SCE enzymes.

WT SCE and SCE K15R can sumoylate model substrate NAF, whereas the active site mutants SCE C94S and SCE C94A cannot. A ten-fold excess of SCE active site mutant proteins SCE C94S or C95A decreases the activity of WT SCE or SCE K15R. Lines 1 to 4 are identical to those of Figure 1.

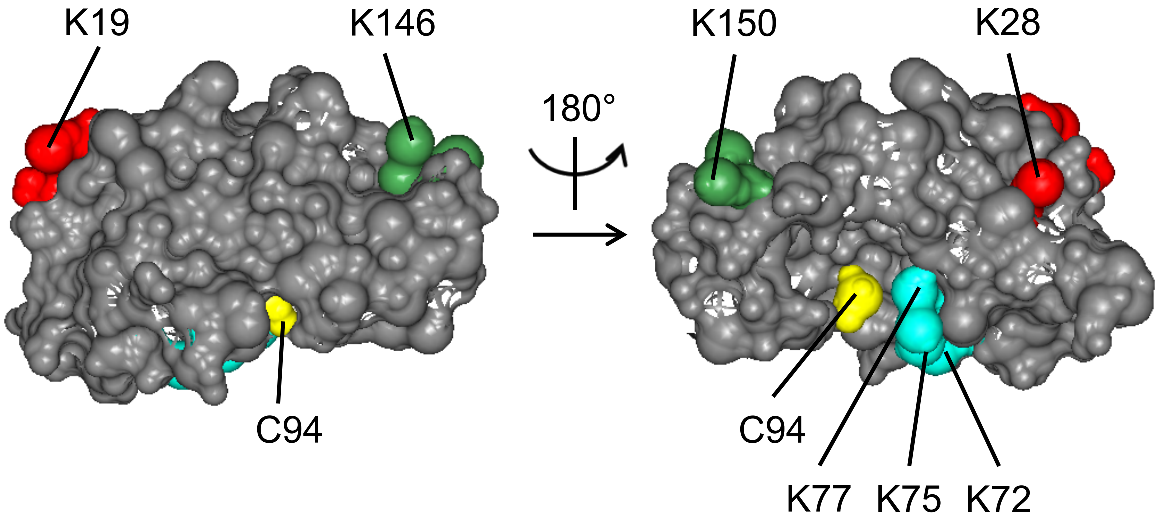

Supplementary Figure 4. Arg residues in the vicinity of the active site of Arabidopsis SUMO conjugating enzyme.

In order to confirm loading of the SCE C94S mutant with SUMO by SUMO activating enzyme, Lys residues 72, 75 and 77 were mutated to Arg. This change prevents potential transfer of SUMO from E1 or SCE C94 to the vicinity of the active site. The K72,75,77R mutations did not prevent formation of a SUMO adduct of SCE1 C94S by SUMO activating enzyme, supporting a transfer to Ser in position 94. Red: Lys 15, 19, 28. Green: Lys 146, 147, 150, 154. Turquois: Lys 72, 75, 77. See also Fig. 2. for legend.

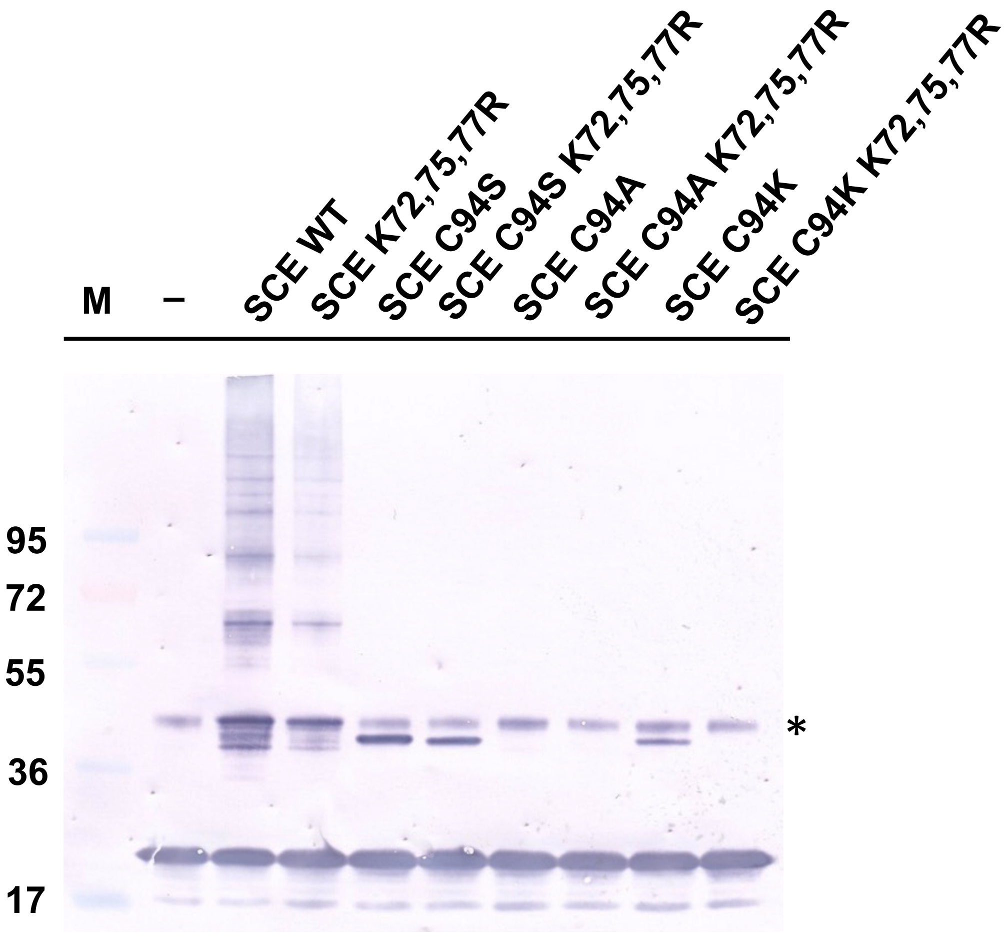

Suppelmentary Figure 5. SUMO conjugated to the active site is not transferred to a nearby Lys residue.
Mutations in the active site Cys residue (C94) abolish activity of SCE, but a replacement by Ser still allows loading with SUMO (see main text). Changing Lys residues K72, K75 and K77, which are adjacent to the active site, to Arg, does not prevent SUMO transfer, indicating that the transfer occurs to the active site Ser, and not to adjacent Lys residues. The asterisk indicates a contaminating protein in the SUMO preparation (also visible in lane –).

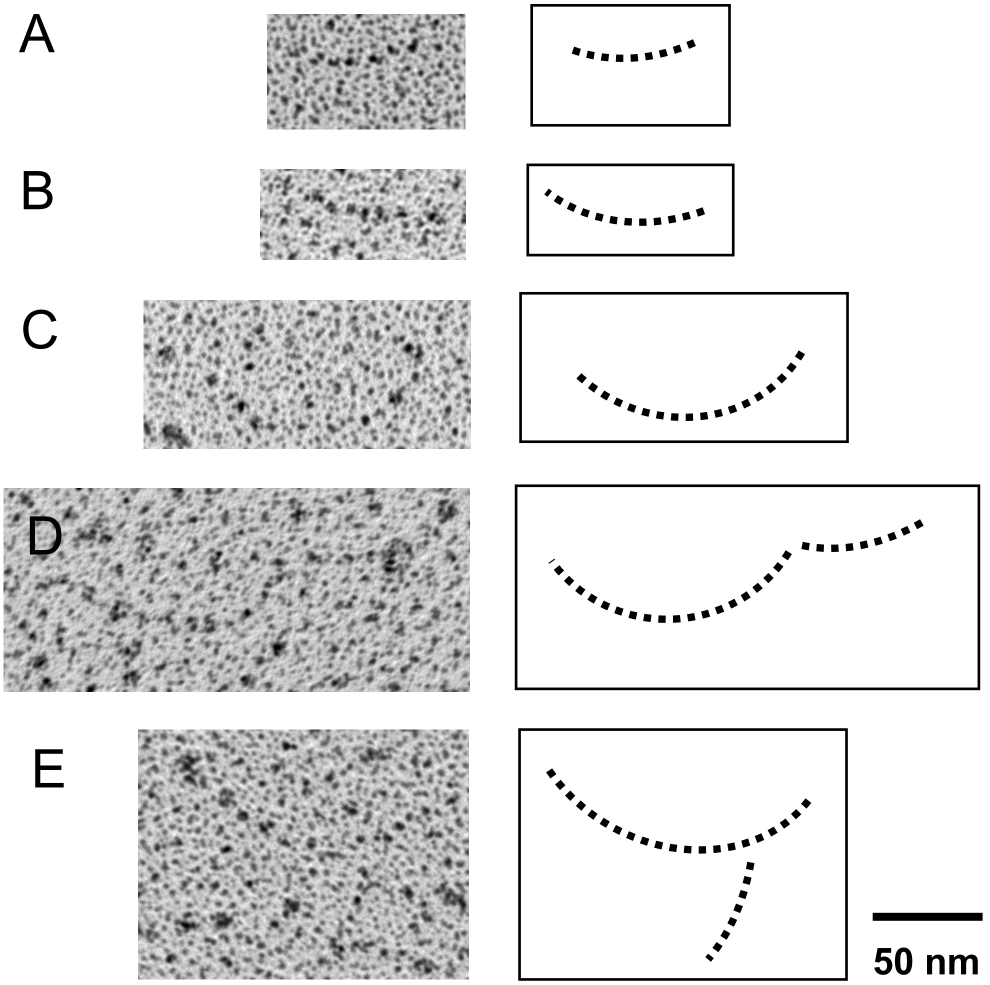

Supplementary Figure 6. Electron microscopy images of SUMO chains after rotary shadowing.

SUMO chains were synthesized *in vitro*, spread on gold grids and subjected to rotary shadowing. Interpretation of the images is to the right. Panel E shows the only instance found that might display a branched chain, indicating that branched SUMO chains are rare if existing at all.

Spplementary Table 1. Oligonucleotides used in this work

Name of oligo	Sequence	Use
1618-SCE1K15R-fwd	CGT TTA GCT GAA GAG AGG AGA TCG TGG AGG AAG AAT CAT	Mutagenesis K15R
1619-SCE1K15R-rev	ATG ATT CTT CCT CCA CGA TCT CCT CTC TTC AGC TAA ACG	Mutagenesis K15R
1965-SCE1K19Rfwd	GAG AGG AGA TCG TGG AGG AGG AAT CAT CCT CAT GGT TTT	Mutagenesis K15,19R
1966-SCE1K19Rrev	AAA ACC ATG AGG ATG ATT CCT CCT CCA CGA TCT CCT CTC	Mutagenesis K15,19R
1967-SCE1K28Rfwd	CCT CAT GGT TTT GTG GCA AGG CCG GAG ACG GGG CAG GAT	Mutagenesis K15,19,28R
1968-SCE1K28Rrev	ATC CTG CCC CGT CTC CGG CCT TGC CAC AAA ACC ATG AGG	Mutagenesis K15,19,28R
1969-SCE3K3Rfw	GAT CCA GTT GAG TAC AGG AGA AGG GTG AGG CTG CAG TCC AGG CAG TAT CCT GCT CTT	Mutagenesis K146,147,150,154R
1970-SCE3K3Rrv	AAG AGC AGG ATA CTG CCT GGA CTG CAG CCT CAC CCT TCT CCT GTA CTC AAC TGG ATC	Mutagenesis K146,147,150,154R
1971-SCE1C94Afw	TAT CCA TCT GGA ACT GTC GCT CTC TCT ATC CTT AAC GAG	Mutagenesis C94A
1972-SCE1C94Arv	CTC GTT AAG GAT AGA GAG AGC GAC AGT TCC AGA TGG ATA	Mutagenesis C94A
1979-SCE1C94Kfw	TAT CCA TCT GGA ACT GTC AAA CTC TCT ATC CTT AAC GAG	Mutagenesis C94K
1980-SCE1C94Krev	CTC GTT AAG GAT AGA GAG TTT GAC AGT TCC AGA TGG ATA	Mutagenesis C94K
2028-SCE1K28Qfwl	CAT CCT CAT GGT TTT GTG GCA CAG CCG GAG ACG GGG CAG GAT GG	Mutagenesis K28Q
2029-SCE1K28Qrvl	CCA TCC TGC CCC GTC TCC GGC TGT GCC ACA AAA CCA TGA GGA TG	Mutagenesis K28Q
2032-SCE1K28Rf2	CAT CCT CAT GGT TTT GTG GCA AGG CCG GAG ACG GGG CAG GAT GG	Mutagenesis K28R
2033-SCE1L28Rr2	CCA TCC TGC CCC GTC TCC GGC CTT GCC ACA AAA CCA TGA GGA TG	Mutagenesis K28R
1656-SCEgdn1	CCC GCC CTC GAG TGA CCT GCA AAA ACT GCT TCC	genomic amplification
1657-SCEgup1	CCC GCC ACT AGT GAA ATA TAT GTA TTT CTA GAT TCA C	genomic amplification
1757-SCEtermup2	TCC AAG CTT TCT ATC TCA ATC	detection of genomic SCE1
1711-SCE889-914	ACG ATA CTC CGA TTA CCC TTC TAA AG	detection of genomic SCE1 and T-DNA insert in *sce5-1*
Salk LBa1	TGG TTC ACG TAG TGG GCC ATC G	detection of T-DNA insert in SCE
1456-BAR_F	AGA AAC CCA CGT CAT GCC AGT TC	detection of T-DNA construct
1619-SCE1K15R-rev	ATG ATT CTT CCT CCA CGA TCT CCT CTC TTC AGC TAA ACG	detection of T-DNA construct
